# Atomic Simulation of Nanoindentation on the Regular Wrinkled Graphene Sheet

**DOI:** 10.3390/ma13051127

**Published:** 2020-03-03

**Authors:** Ruonan Wang, Haosheng Pang, Minglin Li, Lianfeng Lai

**Affiliations:** 1 School of Mechanical Engineering and Automation, Fuzhou University, Fuzhou 350002, Fujian, China; n170220003@fzu.edu.cn (R.W.); m150210010@fzu.edu.cn (H.P.); 2 Fujian Key Laboratory of Medical Instrumentation and Pharmaceutical Technology, Fuzhou University, Fuzhou 350002, Fujian, China; 3 College of Information and Mechanical & Electrical Engineering, Ningde Normal University, Ningde 350900, Fujian, China

**Keywords:** wrinkle, molecular dynamics simulations, graphene, boundary conditions, nanoindentation

## Abstract

Surface landscapes have vague impact on the mechanical properties of graphene. In this paper, single-layered graphene sheets (SLGS) with regular wrinkles were first constructed by applying shear deformation using molecular dynamics (MD) simulations and then indented to extract their mechanical properties. The influence of the boundary condition of SLGS were considered. The wrinkle features and wrinkle formation processes of SLGS were found to be significantly related to the boundary conditions as well as the applied shear displacement and velocity. The wrinkling amplitude and degree of wrinkling increased with the increase in the applied shear displacements, and the trends of wrinkling wavelengths changed with the different boundary conditions. With the fixed boundary condition, the degree of graphene wrinkling was only affected when the velocity was greater than a certain value. The effect of wrinkles on the mechanical characterization of SLGS by atomic force microscopy (AFM) nanoindentation was finally investigated. The regular surface wrinkling of SLGS was found to weaken the Young’s modulus of graphene. The Young’s modulus of graphene deteriorates with the increase in the degree of regular wrinkling.

## 1. Introduction

Since single-layered graphene sheets (SLGS) were prepared for the first time by mechanical exfoliation in 2004 [1], the discovery set off a wave of studies on two-dimensional nanomaterials [2,3,4], and the industrial application of two-dimensional nanomaterials has become an emerging field in today’s world over the past decade [5,6,7,8,9]. In 2007, Meyer et al. [10] first discovered the wrinkling phenomenon on the graphene surface. Two years later, Bao et al. [11] first directly observed and created periodic ripples in SLGS by adopting both spontaneously and thermally generated strains. Further studies revealed that wrinkling has significant impacts on the physical property of graphenes such as electrical [12] and mechanical properties [13,14,15].

Regarding mechanical properties, it has been found that wrinkles have vague effects on the behavior of atomic force microscopy (AFM) nanoindentation. For example, Ruiz-Vargas et al. [13] found that inherent wrinkles appeared on the surface of the graphene sheet prepared by chemical vapor deposition (CVD) where the wrinkles weakened the elastic stiffness of the sheet. Lin et al. [14] reported that under large load, the loading–unloading cycles of graphene displayed a marked increase in stiffness, which may result from the flattening of wrinkles. López-Polín et al. [15] imposed a large enough tension on the SLGS to restrain atomic fluctuations and recorded an increase of graphene’s stiffness. However, the surface features of the graphene in these previous experimental studies were irregular, and no parameters were used to characterize the surface wrinkles. In other words, wrinkles in these studies were often formed on CVD-graphene in an uncontrollable way. Due to the ambiguous surface topography, the effect of the wrinkling properties such as wavelength and amplitude on the mechanical properties of graphenes remain obscure. Therefore, in the present work, molecular dynamics (MD) simulations of AFM nanoindentation were employed to study the wrinkling effect on the mechanical properties of SLGS from a quantitative perspective.

The formation of the wrinkling of SLGS with various structural shapes has been studied by applying different strains based on the molecular dynamics (MD) simulations [16,17,18,19,20,21,22,23]. A sphere tip was used to indent a circular graphene [18] with a simply supported boundary to form the wrinkling as the indentation depth reaches a critical value [17]. Shear strains have been applied to rectangular [16,19,22,23] and toroidal [20] models to form regular wrinkles in graphene, and the boundary condition and loading case have great impacts on the wrinkling morphology. To the best of our knowledge, edge atoms subjected to shear strain were usually fixed or controlled in the rectangular models, and the formed wrinkles were generally distorted near the boundaries. Meanwhile, previous studies on graphene wrinkling have been limited to the investigation of the wrinkling features such as pattern, wavelength, amplitude, and wrinkling process. Very few efforts have been devoted to the effects of regular wrinkles on the indentation behavior of SLGS. Through investigating the propagation process and formation mechanism of graphene, the physics of wrinkled graphene can be better understood. The characteristic parameters and configuration of wrinkles can also be controlled to meet the engineering requirements. The results of the SLGSs’ deflection response to the AFM nanoindentation experiment were used to investigate the influence of the wrinkling phenomenon on the elastic properties of SLGS.

The present paper is focused on the formation of regular wrinkles in SLGS and the effects of these wrinkles on the elastic stiffness of SLGS using MD indentation simulations. The regular wrinkles were first generated by applying shear strains under different boundary conditions. Aside from the simply supported boundary, four other kinds of boundaries were adopted to investigate the effects of boundary conditions on the wrinkle propagation process and formation mechanism. The effects of factors such as atomic displacement and loading velocity on the features of the generated wrinkles were also considered. The effect of the regular wrinkle degree on the Young’s modulus of SLGS was finally investigated.

## 2. Theoretical Model and Methods 

### 2.1. Wrinkle Model

**Figure 1:**
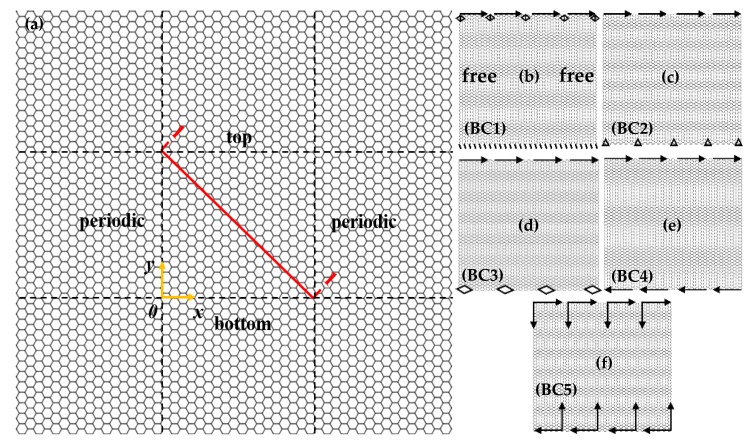
(**a**) The periodic image of the single-layered graphene sheets (SLGS) model, and (**b**–**f**) SLGS with five forms of boundary conditions.

The area of the rectangle SLGS was 11.0604 × 10.6980 nm^2^ in the X–Y plane, and the C–C bond length was about 0.142 nm [1]. The interaction between the carbon atoms of the SLGS was determined by the adaptive intermolecular reactive empirical bond-order (AIREBO) potential, which has accurately described the elastic properties of carbon structures [24,25]. The temperature was set at 0.1 K, which can minimize the interference of thermal fluctuation to the simulation process and has been verified in our previous studies [26,27]. All simulations in this paper were performed under the NVT ensemble in the open source software Large-Scale Atomic/Molecular Massively Parallel Simulator (LAMMPS) [28]. The wrinkling propagation process was observed in OVITO software [29]. In the MD simulations, the periodic boundaries were adopted for the *x*, *y*, and *z* directions to ensure that the density of the system remained constant. Here, the simulation box was deformed only in the y direction, so SLGS with an infinite length in the x direction can be achieved by applying a periodical boundary on the free edges. It is difficult to apply a shear displacement in a periodic box due to the existence of the periodic image atoms, as shown in Figure 1a. It is also impractical for us to construct a micron-sized simulation box due to the high computational expense this would entail.

The graphene sheet was sufficiently relaxed before the application of velocity. The system was relaxed using energy minimization with the conjugate gradient algorithm. The convergence criteria on energy and forces were set to 0.0 and 1.0 × 10^−6^ eV/Å, respectively.

Considering that the fixed boundary generally induces local distortion of wrinkles, in order to generate regular wrinkles throughout the whole model, the edge atoms with four other forms were controlled. Figure 1b–f shows the five boundary conditions, named BC1, BC2, BC3, BC4, and BC5. The degree of freedoms of atoms at the left and right edges were set up to be free for all models. Table 1 lists the degree of freedoms (DOFs) of the top and bottom atoms in these five boundary conditions. These boundary conditions are not intended to produce corresponding wrinkles in actual conditions, but to simulate local areas of real wrinkles. For all boundary conditions, displacements along the x direction were applied on the top atoms to generate the shear strains, denoted by the horizontal arrows. In particular, displacements along the negative x direction were additionally applied on the bottom atoms for BC4 and BC5. More specifically, the top and bottom atoms in the case of BC5 have opposite displacements during the shear deformation. The cross-section 1–1 of the red line in Figure 1a is the diagonal line of the model. The features of the generated wrinkles were characterized by the out-of-plane displacement of atoms in cross-section 1–1. 

### 2.2. Atomic Force Microscopy (AFM) Nanoindentation Model

**Figure 2:**
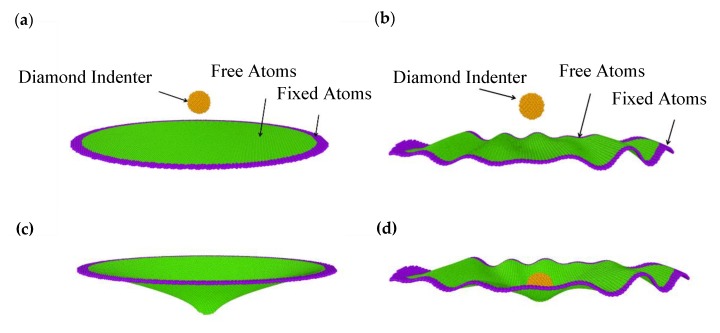
The molecular dynamics (MD) models of the (**a**) perfect and (**b**) wrinkled circular graphene sheets for nanoindentation simulations, and the arbitrary states of indentation on the (**c**) perfect and (**d**) wrinkled graphenes.

In order to characterize the mechanical properties of wrinkled graphenes, circular atomic models were reconstructed from the generated rectangular wrinkled graphenes for the indentation simulations. The schematic representation of the nanoindentation models for perfect and wrinkled circular graphenes is shown in Figure 2. The diameters of the whole circular models were set as 22 nm, and the circular regions colored by the green area modeled the real graphene samples suspended over a hole in the substrate. The diameters of the green circulars were set as 20 nm. The purple atoms were fixed to represent the clamped boundary conditions in real nanoindentation experiments. The size of the spherical diamond indenter was set to be 2 nm and we assumed that the indenter was rigid. It was noted that the ratio of indenter radius to suspended graphene radius was less than 0.1, therefore the point loading model is herein reasonable to be used to accurately characterize the elastic properties of graphenes [30,31,32]. The circular model indented by a point load is generally used for obtaining the elastic stiffness of two-dimensional (2D) nanomaterials, according to the pioneer work of Lee [32], and the larger indenter can result in the relatively high deviation of the Young’s modulus in our previous simulation studies [26].The interaction between the SLGS and the diamond indenter was determined by the Lennard–Jones (LJ) potential, which has been successfully applied in previous works [24,30,33] under the same simulation conditions. In the initial state, the indenter was located 3 nm directly above the center of the graphene sheets, followed by the energy minimization of the perfect graphene membrane, and then pressed down on the graphene sheet at a rate of 0.1 Å/ps. Figure 2c,d show the arbitrary states of indentation on the perfect and wrinkled graphenes. The initial wrinkling state in Figure 2d was obtained from the boundary condition of BC4 at a speed of 0.2 Å/ps.

## 3. Results and Discussions

### 3.1. Characterization of Wrinkles

**Figure 3:**
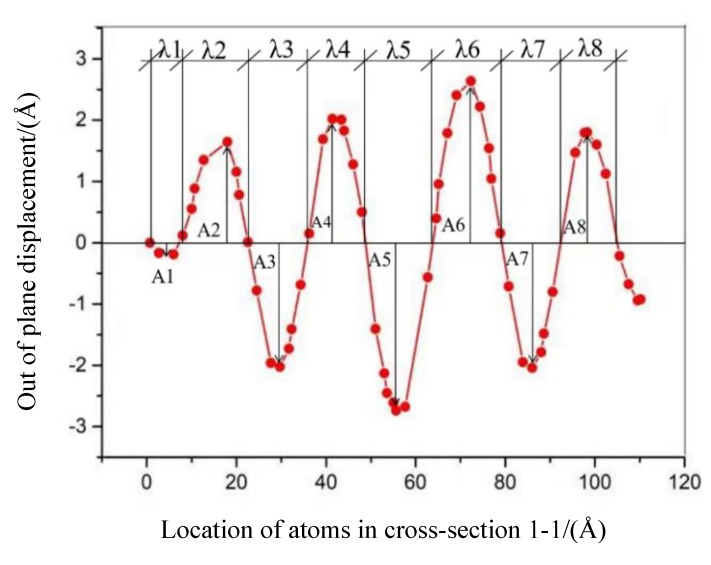
Wrinkling shape in cross-section 1–1 of Figure 1 (BC1 with an applied shear speed of 0.1 Å/ps and a total shear displacement of 10 Å).

Unlike using analytical expression with trigonometric functions, according to continuum modeling for the description of the out-of-plane displacement [21], a simple arithmetic mean expression was adopted to describe the wrinkling characterization, given as [21,34].

(1) $$\eta =\frac{1}{n}\sum_{i=1}^{n}\eta_{i}$$

where *η* denotes a specific characteristic parameter of wrinkles such as wrinkling wavelength *λ*, amplitude *A*, and the ratio of wrinkling amplitude to wavelength *A/λ*. The value of *η* is used to reflect the average value of the wrinkle characteristics of SLGS. The ratio of wrinkling amplitude to wavelength, *A/λ*, is used to describe the degree of the wrinkling. *n* represents the total number of wrinkles, *n* (*i*, *j*), given by the sum of the number of crests, *i*, and the number of troughs, *j*. For example, Figure 3 shows a wrinkling shape in cross-section 1–1 defined in Figure 1a, obtained from the BC1 case under a 1 nm shear displacement. The total number of wrinkles, *n* (*i*, *j*), equals eight (four wrinkling crests and four troughs). The wrinkling wavelength of *λ* is the mean value of *λ*_1_–*λ*_8_, and the wrinkling amplitude of *A* is the mean value of *A*_1_–*A*_8_.

### 3.2. Influence of Loading Velocity on Wrinkling Parameters

**Figure 4:**
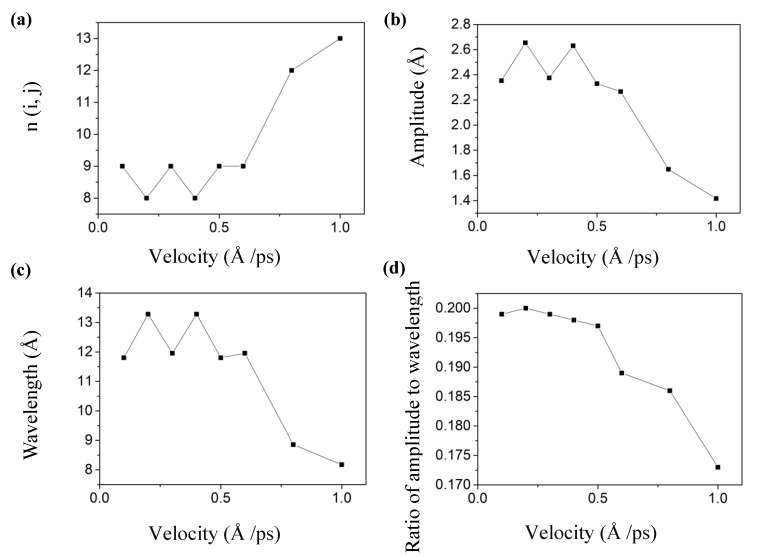
The relationship between the velocity and wrinkling parameters: (**a**) Wrinkling number, (**b**) Amplitude, (**c**) Wavelength, (**d**) Ratio of amplitude to wavelength.

The loading velocity is widely supposed to have an influence on the mechanical behavior of 2D nanomaterials using MD simulations [24,26]. To find the proper loading velocity for forming the wrinkled graphene in our MD simulations, we took a rectangular graphene with the BC1 boundary condition as an example. The total shear displacement of the top edge atoms was set as 15 Å, and a series of loading velocities (0.1, 0.2, 0.3, 0.4, 0.5, 0.6, 0.8, and 1.0 Å/ps) were adopted to investigate the effect of the loading velocities on the wrinkling parameters. Results are shown in Figure 4.

It shows that as the loading velocity increased, the wrinkling amplitude *A*, the wavelength *λ*, and the ratio *A/λ* generally decreased. The wrinkling amplitude *A* and wavelength *λ* significantly increased at *v* = 0.2 Å/ps and *v* = 0.4 Å/ps due to the decreases in the wrinkling numbers, and this sudden decrease influences the mean value of the wrinkling parameters. When the loading velocity was smaller than 0.6 Å/ps, the wrinkling parameters were almost unaffected. However, when the loading velocity was greater than 0.6 Å/ps, the wrinkling parameters will decrease sharply, indicating that the loading velocity has a remarkable influence effect on the wrinkling parameters only when it exceeds a certain value. This result was different from the experimental results of Huang et al. [16], where the loading velocity had little effect on wrinkling parameters. We consider that as the loading velocity increases, the structure enters a higher-order buckling state with a sharp increase in the number of wrinkles. The higher velocity may also influence the consequence; this mechanism will be studied in future work by decreasing the strain rate through a convergence study to eliminate its effect.

It is worth noting that large kinetic energy and atomic displacements may trigger non–trivial anharmonic effects, further affecting both the wrinkling parameters and the Young modulus, even though the calculations are carried out at an ultra–cold temperature of 0.1 K. We plan to investigate the anharmonic effects in future studies and shown in other submissions.

### 3.3. Wrinkle Formation under Different Boundary Conditions

**Figure 5:**
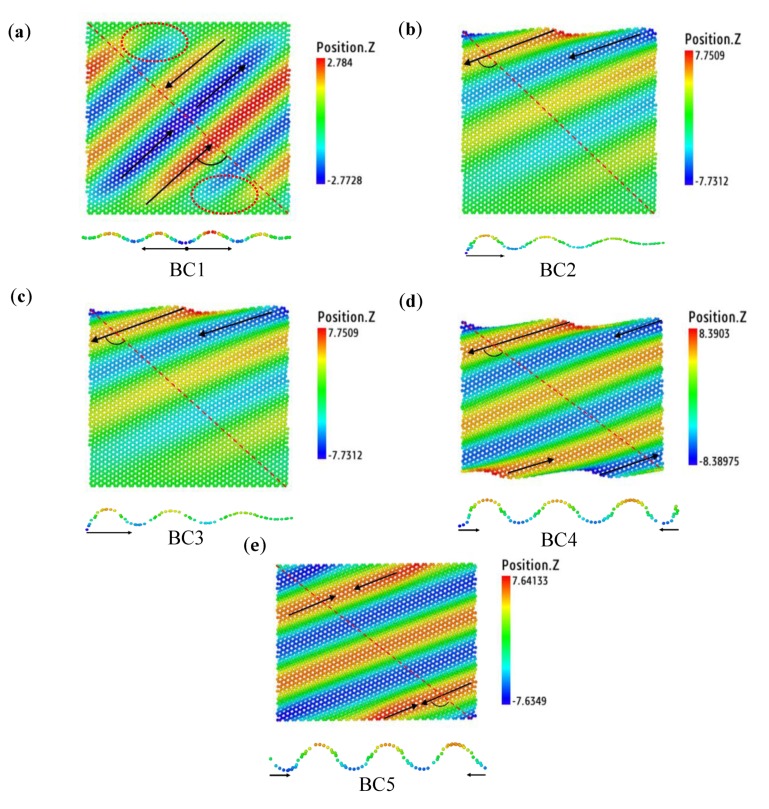
The wrinkle morphologies of graphene sheets under (**a**) BC1, (**b**) BC2, and (**c**) BC3 with a shear displacement of 1 nm applied on the top boundaries, and under (**d**) BC4 and (**e**) BC5 with a shear displacement of 1 nm applied on both the top and bottom boundaries. The shear velocities are 0.1 Å/ps.

The boundary conditions have a significant influence on the wrinkles of graphene [16]. However, only clamped and fixed boundary conditions have been investigated in the previous works, which generally results in local distorted wrinkling morphologies [16,21,34]. In this paper, we used five forms of boundary conditions in an attempt to produce a regular wrinkle throughout the whole rectangular graphene sheet. Figure 5 shows the ultimate morphology of the wrinkles of the SLGS after applying an atomic displacement of 1 nm. The red lines in Figure 5 shows the cross-section 1–1 (shown at the bottom of every subfigure) to calculate the wrinkling parameters. Unlike previous works [16,21,34], the cross-section 1–1 was almost parallel to the propagation direction of wrinkles, as shown in Figure 5, which is expected to contain more information. The black arrows represent the directions of the wrinkling growth. The angle between the wrinkling propagation direction and the cross-section 1–1 is also shown in Figure 5. The evolutions of the wrinkles were observed with OVITO software (see Graphics interchange format (Gif) images of Loading velocities in Supplementary Materials S2).

Under the boundary condition of BC1, wrinkles initially take shapes on the left and right free edges, then propagate to fixed boundaries. The new wrinkles split away from the original ones and are then evenly distributed on the surface of the graphene sheet to produce parallel wrinkles with an angle of roughly 45° to the fixed edges. Similar to previous works [16,21,34], the fixed flat boundary conditions led to the curvature in the wrinkles to gradually reduce down to zero from the center horizon lines of the rectangle to the top and bottom boundaries. The wrinkles distorted near the boundaries as shown in the red circles of Figure 5a. For the boundary condition of BC2, due to the loading displacement applied to the top atoms, the wrinkles initially appeared on the top boundary and then propagated to the free boundary. The new wrinkles were formed due to the extrusion from top atoms. The dynamic formation process can be seen in the Supplementary Materials. The evolution of the wrinkling under BC3 was totally similar to that under BC2, which means that unshackling the horizontal DOF of the bottom atoms does not have any effect on the formation of wrinkles. In other words, the bottom atoms do not move along with the shear displacement applied in the *x* direction, mainly due to the unshackling of the DOF of the top atoms in the y direction and the shear displacement was insufficiently large enough to produce wrinkle shapes. As for the boundary condition of BC4, the wrinkles initially rose from the top (and bottom) edges and gradually spread to the left (and right) edges. Due to the DOFs in the *y* and *z* directions of the top and bottom atoms, the shear deformation induced the rectangular graphene sheet to shrink along the *y* direction and to form more regular wrinkles throughout the whole sheet. Compared with BC4, the wrinkles under BC5 started from all boundaries, top/bottom and left/right edges, and grew to the red line in Figure 5e. In comparison, the wrinkles formed under BC4 and BC5 were more uniform and regular. Therefore, a circular wrinkled graphene was cut out from the center of the rectangular sheet under BC5 and was indented to investigate the elastic properties.

### 3.4. Influence of Shear Displacement on Wrinkling Parameters

In the above section, it was found that 1 nm shear displacement could not induce wrinkles across the whole rectangular sheets for the boundary conditions of BC2 and BC3. Here, we investigated the effect of shear displacement on the formed wrinkles of SLGS. Six shear displacements of 10 Å, 15 Å, 20 Å, 25 Å, 30 Å, and 35 Å were used at the loading speed of 0.1 Å/ps.

**Figure 6:**
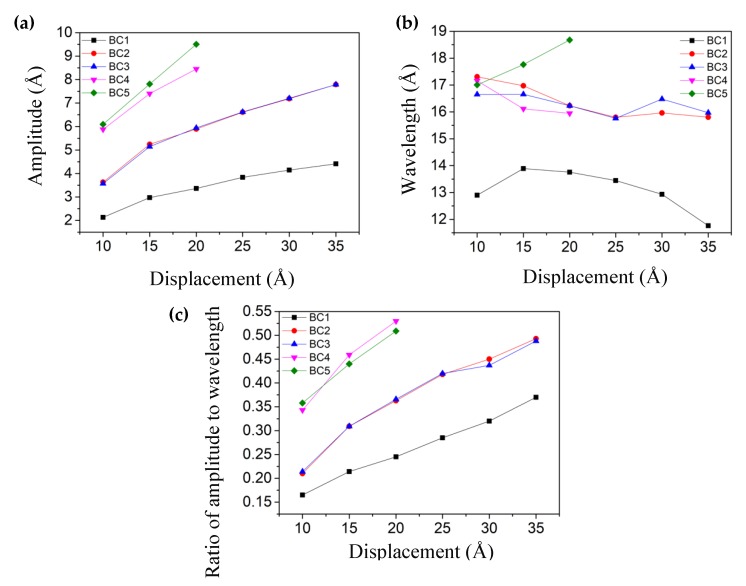
The relationship between the applied shear displacement and the wrinkling parameters: (**a**) Amplitude, (**b**) Wavelength, (**c**) Ratio of amplitude to wavelength, under five boundary conditions (BC1, BC2, BC3, BC4, and BC5).

Figure 6 shows the wrinkling parameters including amplitude *A*, wavelength *λ*, and the ratio of amplitude to wavelength *A/λ* varying with the total shear displacement. It shows that the wrinkling amplitude *A* and ratio *A/λ* (amplitude to wavelength) increased over the shear displacement under all five boundary conditions. Under the former four boundary conditions, the wavelength *λ* gradually decreased as the displacement increased. The forming mechanism of the wrinkling of graphene has been revealed based on elasticity theory [16], where the out-of-plane deformations or wrinkles are enhanced to release the strain energy as the loading displacement increases. It can be confirmed by the increase in the wrinkle number by increasing the shear displacement (see Table S1 in Supplementary Materials). However, for the boundary condition of BC5, the wavelength of *λ* increased at the larger displacements. It can be attributed to the shrinkage of winkled SLGS in the *y* direction and the enlargement of the angle between the wrinkles and the cross-section 1–1. Specifically, with the increase of atomic displacements, the number of wrinkles remained unchanged and the angle between the wrinkles and the cross-section 1–1 kept increasing (see Table S1 in Supplementary Materials). It revealed that the wavelength varied under different boundary conditions. The order of the values of the wrinkling amplitude *A* and the ratio *A/λ* at the same loading displacement was BC4 and BC5 > BC2 and BC3 > BC1, basically due to the different boundary conditions.

### 3.5. Effect of Wrinkles on Young’s modulus of Graphene during Nanoindentation

**Figure 7:**
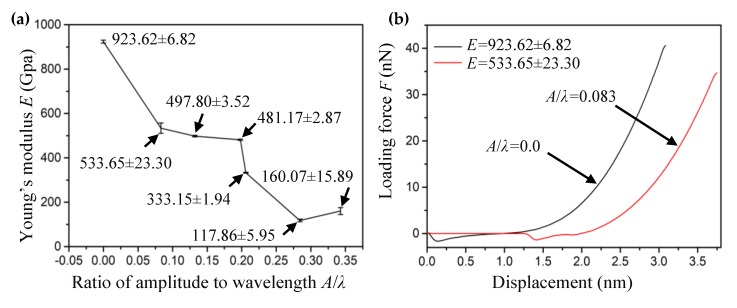
(**a**) The Young’s modulus (*E*) of wrinkled graphene versus the ratio of *A*/*λ*. (**b**) The loading force–displacement curves of graphene (*A*/*λ* = 0 and *A*/*λ* = 0.083).

To reveal the influence of graphene wrinkling on the behavior of AFM nanoindentation or the mechanical properties, we indented the perfect and wrinkled circular graphenes to compare their Young’s moduli. Herein, the loading force *F* at the center of mass of the indenter and the vertical position of the indenter relative to the substrate *Z* were recorded to obtain a classic force-displacement curve. The revised numerical method proposed by Lin et al. [14] was adopted to extract the Young’s modulus, since the revised method can effectively eliminate the impact of choosing different zero-displacement point (ZDP) on the fitting Young’s modulus. The fitting formula proposed by Lin [14] is described as follows:

(2) $$F=f_{0}-\sigma_{0}^{2D}\pi \delta_{0}-\frac{E^{2D}q^{3}\delta_{0}^{3}}{a^{2}}+\left(\sigma_{0}^{2D}\pi +\frac{3E^{2D}q^{3}\delta_{0}^{2}}{a^{2}}\right)Z-\frac{3E^{2D}q^{3}\delta_{0}}{a^{2}}Z^{2}+\frac{E^{2D}q^{3}}{a^{2}}Z^{3}$$

where $f_{0}$ and $\delta_{0}$ correspond to the measured force and associated vertical position of the tip, respectively. These parameters are free parameters and can be deduced by fitting the curve using Equation (2). Equation (2) is used for the point loading model (whose ratio of indenter radius to suspended graphene radius is less than 0.1). $E^{2D}$ is the 2D Young’s modulus of the SLGS, *a* is the sheet’s radius. *q* = 1/(1.05−0.15*v*−0.16*v*^2^) is a constant where *v* = 0.165 [35] is the Poisson’s ratio of the graphene sheet. The *E* = $E^{2D}/t$ is the 3D Young’s modulus with the thickness of *t =* 0.335 nm [32]. By fitting the curve using Equation (2), the Young’s modulus (*E* = 923.62 ± 6.82 GPa) for the perfect graphene sheet was derived, which was close to the result of the experimental tests (1 TPa) by Lee et al [32,35]. As shown in Figure 2, a circular wrinkled graphene with a 20 nm diameter was obtained from the rectangular graphene sheet with a 30 × 30 nm^2^ area subjected to different shear displacements under the boundary condition of BC4. To ensure that the simulation was carried out within the elastic deformation, the loading and unloading process was also performed to record the curve data of loading force and indenter displacement. The relationship between shear strain (*γ*) and the ratio of wrinkling amplitude to wavelength (*A*/*λ*) can be expressed as *A*π/*λ* = (2*γ* − 2*νγ*)^1/2^ [36]. Therefore, the *A*/*λ* is used to describe the degree of the wrinkling under the shear deformation [13]. The fitting Young’s modulus (*E*) corresponding to the ratio of *A*/*λ* based on Equation (2) is shown in Figure 7a.

It can be seen that the wrinkling significantly decreased the Young’s modulus of the SLGS, which is consistent with the experimental results obtained by Ruiz-Vargas et al. [13]. The mechanism has been revealed based on a spring model; as the spring constant of the wrinkles is much smaller than that of the graphene, therefore the energy needed for flattening wrinkles is less than that for stretching the graphene membrane [13]. It is worth noting that the wrinkles with a different ratio of amplitude to wavelength had different levels of impact on the elastic stiffness of wrinkled graphenes. In the range of *A*/*λ* around 0.1 and 0.2, the Young’s modulus decreased gently, while at the value of 0.35, the Young’s modulus unexpectedly increased, perhaps due to the high density of the wrinkles. In order to illustrate the influence of the wrinkling phenomenon on the elastic properties of the SLGS, the load–displacement curves in two representative cases (the perfect graphene membrane with *A/λ* = 0 and the wrinkled graphene membrane with *A/λ* = 0.083) are shown in Figure 7b. In the initial state, there is no force between the indenter and graphene membranes. With the indenter pressed down on the graphene membranes, the force between the indenter and graphene membranes changes from the attractive force to repulsive force. This shows that the perfect graphene membrane has a larger elastic modulus. 

## 4. Conclusions

In summary, five boundary conditions were considered to simulate the local areas of real wrinkles. By investigating the wrinkling deformation of the SLGS, we reached the following conclusions. First, the wrinkling parameters of *A*, *λ*, and *A*/*λ* generally decrease as the velocity increases. The loading velocity influences the wrinkling parameters when it exceeds a certain value, which is different to the experiment results in previous studies. Second, the number of wrinkles becomes intensive and stable as the atomic displacement increases. The wrinkling amplitude (*A*) and ratio of amplitude to wavelength (*A*/*λ*) increased over the atomic displacement (*d*) under all five boundary conditions. Third, under different boundary conditions, the evolution of the wrinkling formation process and wavelength *λ* are different. The forming mechanism of the wrinkling of graphene was revealed based on elasticity theory [16]. Moreover, the effect of wrinkles on the mechanical characterization of graphene was investigated, where the surface wrinkling significantly decreased the Young’s modulus of the graphene sheet at different levels.

The research results have important scientific significance for the effective control and rational application of the surface morphology of wrinkling graphene sheets and the accurate characterization of graphene’s intrinsic properties. 

## Figures and Tables

**Table 1:** The degree of freedoms (DOFs) under five boundary conditions. (U*x*, U*y* and U*z* represent the DOFs along the *x, y*, and *z* directions, respectively.).

Boundary Conditions	Top Boundary	Bottom Boundary
BC1	U*x* is free, and other DOFs are fixed	all DOFs are fixed
BC2	all DOFs are free	U*z* is free, and other DOFs are fixed
BC3	all DOFs are free	U*y* is fixed, and other DOFs are free
BC4	all DOFs are free	all DOFs are free
BC5	all DOFs are free	all DOFs are free

## Supplementary Materials

The following are available at https://www.mdpi.com/1996-1944/13/5/1127/s1. Table S1: Wrinkling parameters under five boundary conditions; Graphics interchange format (Gif) images in S2: Boundary conditions (BC1.gif, BC2.gif, BC3.gif, BC4.gif and BC5.gif); Graphics interchange format (Gif) images in S2: Loading velocities (v1.gif, v2.gif, v3.gif, v4.gif, v5.gif, v6.gif, v7.gif and v8.gif). The v1, v2, v3, v4, v5, v6, v7 and v8 in S2 represent the loading velocities of 0.1, 0.2, 0.3, 0.4, 0.5, 0.6, 0.8, and 1.0 Å/ps, respectively.
