# Supplementary material for: Atomic Simulation of Nanoindentation on the Regular Wrinkled Graphene Sheet
Source: Materials (Basel). 2020 Mar 3;13(5):1127. doi: 10.3390/ma13051127 (PMC7084966; doi:10.3390/ma13051127)
Supplement: Supplementary file 1 [file materials-13-01127-s001.zip › materials-723913- -2nd revised - SP/supplementary files/Supplementary Material 1 .docx]

Supplementary Material 1

Atomic Simulation of Nanoindentation on the Regular Wrinkled Graphene Sheet

Ruonan Wang ^1,‡^, Haosheng Pang ^1,‡^, Minglin Li ^1,2,^* and Lianfeng Lai ^3,^*

^1^	School of Mechanical Engineering and Automation, Fuzhou University, Fuzhou, Fujian 350002, China; n170220003@fzu.edu.cn (R.W.); m150210010@fzu.edu.cn (H.P.)

^2^	Fujian Key Laboratory of Medical Instrumentation and Pharmaceutical Technology, Fuzhou University, Fuzhou, Fujian 350002, China

^3^	College of Information and Mechanical & Electrical Engineering, Ningde Normal University, Ningde, Fujian 350900, China

*****	Correspondence: liminglin@fzu.edu.cn (M.L.); lailianfeng82@163.com (L.L.)

^‡^	These authors contributed equally to this work.

Received: 03 February 2020; Accepted: 29 February 2020; Published: date

**Table S1.** Wrinkling parameters under five boundary conditions.

| **BC1** | **Displacement (Å)** | **Angle (°)** | **n（i，j）** |
| --- | --- | --- | --- |
|  | 10 | 99.37 | 7（4,3） |
|  | 15 | 99.37 | 7（4,3） |
|  | 20 | 99.47 | 7（4,3） |
|  | 25 | 99.68 | 7（4,3） |
|  | 30 | 99.52 | 8（4,4） |
|  | 35 | 100.18 | 8（4,4） |
| **BC2** | **Displacement (Å)** | **Angle (°)** | **n（i，j）** |
|  | 10 | 122.036 | 6（3,3） |
|  | 15 | 124.7 | 7（3,4） |
|  | 20 | 129.4 | 7（3,4） |
|  | 25 | 135.52 | 7（3,4） |
|  | 30 | 139.5 | 7(3,4) |
|  | 35 | 146.29 | 7(3,4) |
| **BC3** | **Displacement (Å)** | **Angle (°)** | **n（i，j）** |
|  | 10 | 122.46 | 6（3,3） |
|  | 15 | 124.45 | 7（3,4） |
|  | 20 | 129.44 | 7（3,4） |
|  | 25 | 135.24 | 7（3,4） |
|  | 30 | 140.96 | 7（3,4） |
|  | 35 | 147.16 | 7（3,4） |
| **BC4** | **Displacement (Å)** | **Angle (°)** | **n（i，j）** |
|  | 10 | 131.84 | 6(3,3) |
|  | 15 | 143.14 | 7(3,4) |
|  | 20 | 156.01 | 7(3,4) |
| **BC5** | **Displacement (Å)** | **Angle (°)** | **n（i，j）** |
|  | 10 | 126.012 | 6（3,3） |
|  | 15 | 130.963 | 6（3,3） |
|  | 20 | 136.995 | 6（3,3） |

|  | © 2020 by the authors. Submitted for possible open access publication under the terms and conditions of the Creative Commons Attribution (CC BY) license (http://creativecommons.org/licenses/by/4.0/). |
| --- | --- |
